# Examination of sexual functions and depressive symptoms among infertile and fertile women

**DOI:** 10.12669/pjms.35.5.615

**Published:** 2019

**Authors:** Selda Ozturk, Hatice Kahyaoglu Sut, Leyla Kucuk

**Affiliations:** 1 Selda Ozturk, MSC, Department of Nursing, Trakya University Health Science Faculty, Edirne, Turkey; 2 Hatice Kahyaoglu Sut, Associate Professor, Department of Nursing, Trakya University Health Science Faculty, Edirne, Turkey; 3 Leyla Kucuk, Professor, Department of Nursing, Mental Health and Psychiatry Nursing, Istanbul University-Cerrahpasa Florence Nightingale Faculty of Nursing, Istanbul, Turkey

**Keywords:** Depressive symptom, Infertility, Sexual dysfunctions, Women

## Abstract

**Objective:**

To examine the sexual functions and depressive symptoms of infertile and fertile women.

**Methods:**

This study was conducted between October 2015 and April 2016 using a descriptive, cross-sectional and comparative design. The sample of this study consisted of 96 infertile and 96 fertile women. The data were collected using an information form, the Beck Depression Inventory and the Index of Female Sexual Function. The data were analyzed The Mann-Whitney U test, chi-square test, and Spearman’s correlation analysis.

**Results:**

The rate of sexual dysfunction (87.5% vs. 69.8%) and the Index of Female Sexual Function total score (31.8 ± 7.8 vs 35.7 ± 6.3) were significantly higher in infertile women than fertile women (p=0.003, p<0.001, respectively). The sexual satisfaction and discomfort during sexual intercourse subscales of the Index of Female Sexual Function were significantly lower among infertile women than fertile women (p<0.001 for all); however, no significant difference was observed in the sexual intercourse/libido score of the Index of Female Sexual Function between infertile and fertile women (p=0.590). The correlation coefficients between the Beck Depression Inventory total score and the total and subscale scores of the IFSF did not significantly differ between infertile and fertile women (p>0.05 for all).

**Conclusion:**

The sexual dysfunction rate among infertile women was higher than that among fertile women. Sexual functions decreased when depressive symptoms increased for both infertile and fertile women.

## INTRODUCTION

Infertility is the failure of a couple to achieve pregnancy after at least one year of regular unprotected sexual intercourse.^1^,^2^ According to the World Health Organization, the rate of infertile couples around the world is approximately between 8 and 12%.^3^ In a study conducted in Turkey by the Ministry of Health in 2014 reported that 4.8% of 6364 women were diagnosed with infertility.^4^

Increased infertility due to various factors all over the world has negative effect on the desire and sexual function of the couples and the necessity of sexual intercourse in planned the times, and the couple causes the sexual problems to increase.^5^ In their studies conducted on infertile couples, Shindel et al. (2008) detected 11% and 28% decreases in libido among men among women, respectively.^6^ Another study determined that 40% of infertile women had sexual dysfunction.^7^ Infertility causes different emotional reactions in couples. In the case of infertility, negative effects such as depression, stress, sensitivity in personal communication and loneliness can occur among couples.^6^ The couples’ loss of pleasure and interest in marriage and sexual intercourse combined with the lack of a desired pregnancy cause intense depressive symptoms.^8^ Begum and Hasan compared the levels of depression among healthy and infertile groups and determined that depression was higher among the latter group.^9^ After applying assisted reproduction techniques, Dilek and Beji determined that women experience more severe changes in sensation than men.^10^

The relationship between infertility and both sexual functions and mental state is bidirectional. Karlidere et al.^11^ found a significant and linear relationship between depressive symptoms and sexual functions in infertile couples. Kabil et al.^12^ reported that the prevalence of depression among infertile women with sexual dysfunction was 29.2%, and that of depression among infertile women without sexual dysfunction was 4.3%.

Sexual functions and depressive symptoms are frequently disregarded during the process of infertility treatment because health professionals generally focus on conception. This study examined between sexual functions and depressive symptoms in infertile and fertile women and the results of this study will both guide the health professionals who work at fertility clinics and contribute to the literature on this topic.

## METHODS

The study was conducted using descriptive, cross-sectional and comparative design to examine sexual functions and depressive symptoms among infertile and fertile women. The research data were collected at the assisted reproduction techniques center (infertile group) and gynecology polyclinic (fertile group) of the health research and application center of a university between October 2015 and April 2016. Convenience sampling was used. The sample consisted of 192 women in total (96 infertile, 96 fertile), with a test power of 0.80, an error margin of 5% (0.05), and a confidence level of 95%.

The inclusion criteria for the infertile women were as clinical diagnosis of infertility, history of infertility treatment for at least six months, no history of a chronic disease requiring hospitalization at the clinical level, no self-reported psychiatric diagnosis before infertility treatment, no communication problems and volunteered to participate in the study. The inclusion criteria for the fertile women were as no clinical diagnosis of infertility, no history of a life-threatening chronic disease, no diagnosis of any psychiatric disorder, no communication problems, volunteered to participate in the study.

The data were collected using an information Form prepared by the researchers, the Beck Depression Inventory (BDI), and the Index of Female Sexual Function (IFSF). The information form was prepared by the researchers after reviewing the literature.

Beck Depression Inventory (BDI) objectively measures the degree of somatic, emotional, cognitive and motivational symptoms observed in depression. Low scores indicate few depressive symptoms, whereas high scores indicate many depressive symptoms. In the Turkish adaptation study of BDI, a cut-off score was reported as 17 (17 or higher is depressive symptoms; 17 or lower no depressive symptoms.^13^

Index of Female Sexual Function (IFSF) investigates the sexual functions of women over the last month. It has three sub-dimensions: discomfort during sexual intercourse, frequency of sexual intercourse/libido and sexual satisfaction. An increase in the average total score of the scale is regarded as an increase in sexual function, whereas a decrease is regarded as a decline in sexual function.^14^

The reliability of the BDI and IFSF were examined using reliability analyses. The results were expressed means and standard deviations or as numbers (%). To determine whether differences existed between infertile and fertile women with regard to the quantitative values, Student’s t-test and the Mann-Whitney U test were used. The chi-square test was used to compare the categorical data between infertile and fertile women. Spearman’s correlation analysis was used to examine the relationship between the BDI and IFSF scores among infertile and fertile women, and Fisher’s Z test was used to compare these correlation coefficients between infertile and fertile women. A value of p<0.05 was accepted as significant.

### Ethical approval

The ethics approval was obtained from Trakya University, Faculty of Medicine, Scientific Research Ethics Committee for the ethical conformity of the study (No. TUTF-BAEK 2015/176). The Trakya University Ethics Committee approved the implementation of this study in Edirne, Turkey on September 03, 2015 (TUTF-BAEK 2015/176). All of the study procedures involving human participants were performed in accordance with the ethical standards of the institution and/or national research committee and complied with the 1964 Declaration of Helsinki and its later amendments or comparable ethical standards. Before data collection, the participants were informed about the aim of the study, and their informed consent was obtained. Patient identity information was not included on the data forms.

## RESULTS

The infertile and fertile women were similar in terms of age, age at marriage, family structure and employment status (p>0.05) (Table-I). Average IFSF total and sub-dimension scores among infertile and fertile women are presented in Table-II. The IFSF total score for infertile women and fertile women demonstrating a significant difference between these groups (p<0.001) (Table-II). Sexual functions incidence of infertile and fertile women values significantly differed (p=0.003).

**Table I:** Sociodemographic characteristics of the infertile and fertile women.

	Infertile women (n=96)	Fertile women (n=96)	
Age	31.1 ± 5.1	32.5 ± 5.2	Z:-1,836; p=0.066
Age at marriage	24.2 ± 5.1	22.9 ± 3.9	Z:-1,574; p=0,116
***Family structure***			
Nuclear family	78(81.2%)	85(88.5%)	χ2:1,990; p=0,158b
Extended family	18(18.8%)	11(11.5%)	
***Educational status***			
≤8 years	46(47.9%)	18(18.8%)	χ2:18,375; p<0,001^b^
>8 years	50(52.1%)	78(81.2%)	
***Employment status***			
Housewife	53(55.2%)	45(46.9%)	χ2:1,334; p=0,248^b^
Working	43(44.8%)	51(53.1%)	

Mean ± standard deviation, n (%), χ2= Chi-Square Test (a: Continuity Correction, b: Pearson Chi-Square; c: Fisher Exact Test); Z=Mann-Whitney U.

**Table II:** Comparative IFSF total and sub-dimension scores and distribution of sexual dysfunction rate among infertile and fertile women.

	Infertile Women (n=96)	Fertile Women (n=96)	
***IFSF total score and Sub-dimensions***			
Total	31.8 ± 7.8	35.7 ± 6.3	Z=-3,821; p=<0,001
Sexual satisfaction	3.4 ± 1.0	4.2 ± 0.8	Z=-4,799; p<0.001
Sexual intercourse frequency/libido	3.1 ± 0.7	3.2 ± 0.8	Z=-0,538; p=0.590
Discomfort during sexual intercourse	4.3 ± 1.1	5.2 ± 1.1	Z=-6,721; p<0.001
Sexual Dysfunction	84(87.5%)	67(69.8%)	χ2= 8,963; p=0.003

Mean ± standard deviation, n (%), Z: Mann Whitney U; χ2= Chi-Square Test

Infertile women and fertile women the BDI total scores did not significantly differ (p=.247). Depressive symptoms were identified and these values did not significantly differ (p=0.091) (Table-III).

**Table III:** Comparative of the BDI scores and the presence of depressive symptoms among infertile and fertile women.

	Infertile Women (n=96)	Fertile Women (n=96)	
BDI total score	11.5 ± 9.7	9.9 ± 9.0	Z=-1,158; p=0.247
Depressive symptoms (≥17)	28(29.2%)	18(18.8%)	χ2=2,859; p=0.091
No depressive symptoms (<17)	68(70.8%)	78(81.2%)	

Mean ± standard deviation; n (%), Z: Mann Whitney U, χ²= Chi-Square Test.

The total IFSF score as well as the sexual intercourse frequency/libido, sexual satisfaction and discomfort during sexual intercourse sub-dimension scores decreased as the BDI scores of infertile women increased. No significant difference was found with regard to the correlation coefficients between infertile and fertile women when the BDI total score and the total and sub-dimension scores of the IFSF scale were compared (p>0.05 for all) (Table-IV).

**Table IV:** Relationship between the BDI total score and the FSFI total and sub-dimension scores among infertile and fertile women.

			IFSF			
			Discomfort during sexual intercourse	Sexual satisfaction	Sexual intercourse frequency/libido	Total score
Infertile women	BDI	r_s_	-0.268	-0.220	-0.289	-0.325
		p	0.008	0.025	0.004	0.001
Fertile women	BDI	r_s_	-0.315	-0.392	-0.369	-0.412
		p	0.002	<0.001	<0.001	<0.001
Comparison of correlation coefficients		p	0.726	0.193	0.540	0.491

r_s:_ Spearman’s correlation analysis.

## DISCUSSION

Infertility is a significant health problem with biological, psychological and social dimensions. The failure to become pregnant can cause sexual dysfunction and depression among individuals.^11^

This study determined that infertile women (87.5%) experienced sexual dysfunction more than fertile women (69.8%). Aggarwal, Mishra, and Jasani that infertile women (63.7%) showed more sexual dysfunction than fertile women (46%).^15^ In a meta-analysis, Mendonca et al. reported that the prevalence of sexual dysfunction was higher in infertile women than fertile women.^16^ Pan^17^ and Czyzkowska, Awruk, and Janowski^18^ found that the sexual function of infertile women was worse than that of fertile women, whereas Aggarwal et al. 15, Zare et al. 5 and Drosdzol and Skrzypulec^19^ did not find any difference between infertile and fertile women with regard to the sexual function. Sexuality is regarded as only for reproductive purposes and as a duty in infertile women, planned sexual intercourse, the sense of guilt, and the complex and interventional diagnoses and treatment approaches applied for assisted reproduction lead to sexual dysfunction among infertile women.^11^ Couples might experience sexual dysfunctions during the diagnosis and treatment period of infertility because the sexual sharing and communication between couples are solely based on conception, and intercourse is only performed for reproduction purposes.

Findings of current study demonstrated that the sexual satisfaction that infertile women get from sexual intercourse is significantly lower than that reported by fertile women. Studies of Turan et al.^8^ and Poornowrooz et al 20 demonstrated that the sexual satisfaction of infertile women was worse than that of fertile women. The present study revealed that infertile women experience significantly more discomfort during sexual intercourse (e.g., dyspareunia) than fertile women. Turan et al. 8 reported similar results. No significant difference was found between infertile and fertile women with regard to the frequency of sexual intercourse/loss of libido. Mendonca et al. (2017)^16^ and Poornowrooz et al.^20^ found similar results. Infertile women might think that infertility is a significant problem for the continuation of their marital lives, and communication problems can occur between couples. Communication problems between couples might disrupt sexual function by negatively affecting their sexual lives, which is among the most basic elements of marriage.

The current study determined that infertile women have more depressive symptoms than fertile women; however, this difference was not significant. Taskin et al. obtained similar results.^21^ However, Rao et al.^22^ reported that depressive symptoms were more common among infertile women. The depressive symptoms observed among infertile women are associated with uncertainty about the future, not knowing the duration of the fertility treatment, the possibility of an unsuccessful treatment, the economic burden of the treatment, the pressure of society, worries about the treatment process and techniques, and fears about the continuation of fertility.^2^,^21^

The current study determined that infertile women with depressive symptoms experience more sexual dysfunction; moreover, sexual functions become significantly worse as depressive symptoms increase in fertile women. Kabil et al. 12 studied infertile women and reported that depression was more prevalent among those with sexual dysfunction. Furthermore, these authors argued that depression should be regarded as the reason for sexual dysfunction rather than the reason for women’s infertility. Mert and Ozen 23 investigated the relationship between depression and the sexual function of fertile women and did not find any difference. Women can feel sexually insufficient because of infertility, and depressive symptoms are often observed as a result of the loss of satisfaction and interest in the marriage and sexual intercourse.^24^ Infertile women experience childlessness-related psychosocial problems and stress, become exposed to stigmatization in society, experience a sense of loss, and experience the psychological effects of the assisted reproduction treatment process.^25^

### Limitations of the study

The study data were obtained from the infertile women who was treated in vitro fertilization center of a single state university hospital in Edirne. It is difficult to generalize the results of the study. Further studies with bigger sample size are recommended.

## CONCLUSION

This study found that the incidence of sexual dysfunction among infertile women was higher than that among fertile women. Sexual intercourse/libido levels were similar regardless of fertility; however, levels of sexual satisfaction and discomfort during intercourse were worse among infertile women than fertile women. Furthermore, a decrease in sexual functions was associated with an increase in depressive symptoms among both infertile and fertile women. According to these results, infertile women has more tendency to sexual dysfunctions and depressive symptoms than fertile women. In this context, it is important for the health professionals who work with infertile women to consider this information. Psychosocial evaluations of these women should be performed on a routine basis, and they should be advised to seek support from mental health professionals if required.
